# Supplementary material for: Chiral Recognition of Homochiral Poly (amidoamine) Dendrimers Substituted with R- and S-Glycidol by Keratinocyte (HaCaT) and Squamous Carcinoma (SCC-15) Cells In Vitro
Source: Polymers (Basel). 2021 Mar 27;13(7):1049. doi: 10.3390/polym13071049 (PMC8037736; doi:10.3390/polym13071049)
Supplement: Supplementary file 1 [file polymers-13-01049-s001.pdf]

Supplementary materials

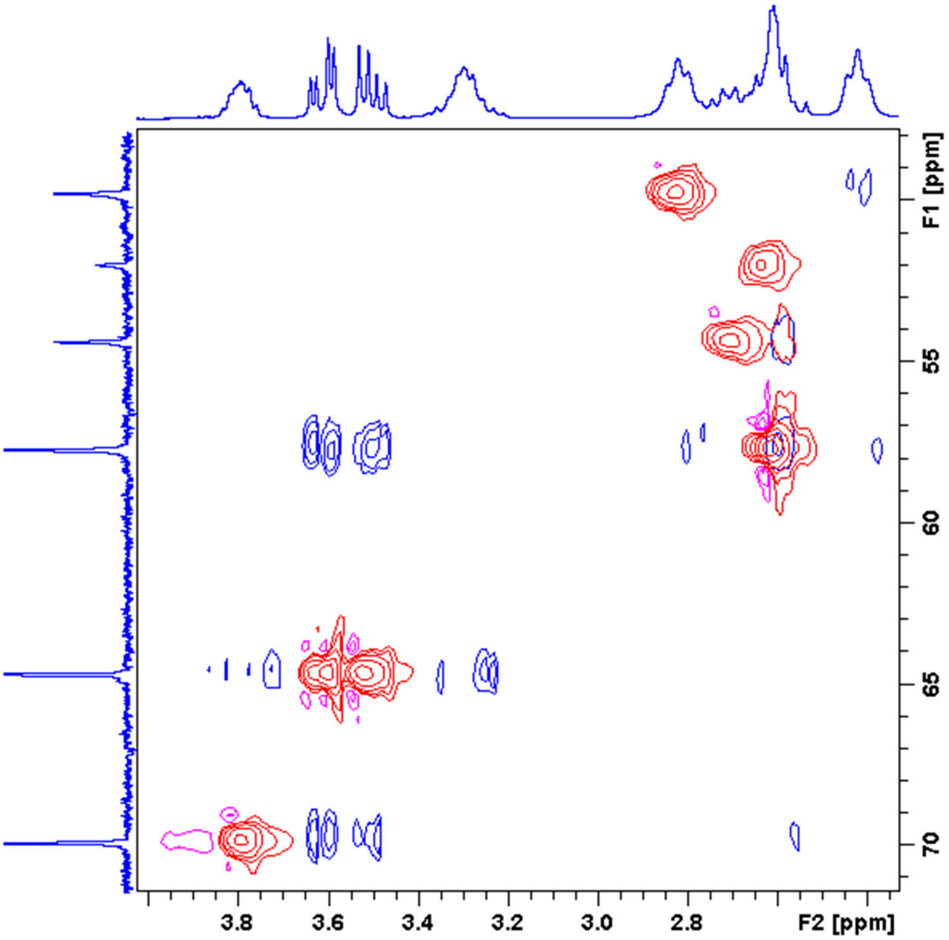

Figure S1. Combined HSQC and HMBC map for **G2**^{32Sgl} Spectra were taken in D_2O. One-bond coupling peaks are shown in red, while relevant longer range (two-bond ^{1}H-^{13}C coupled) peaks are drawn in blue.

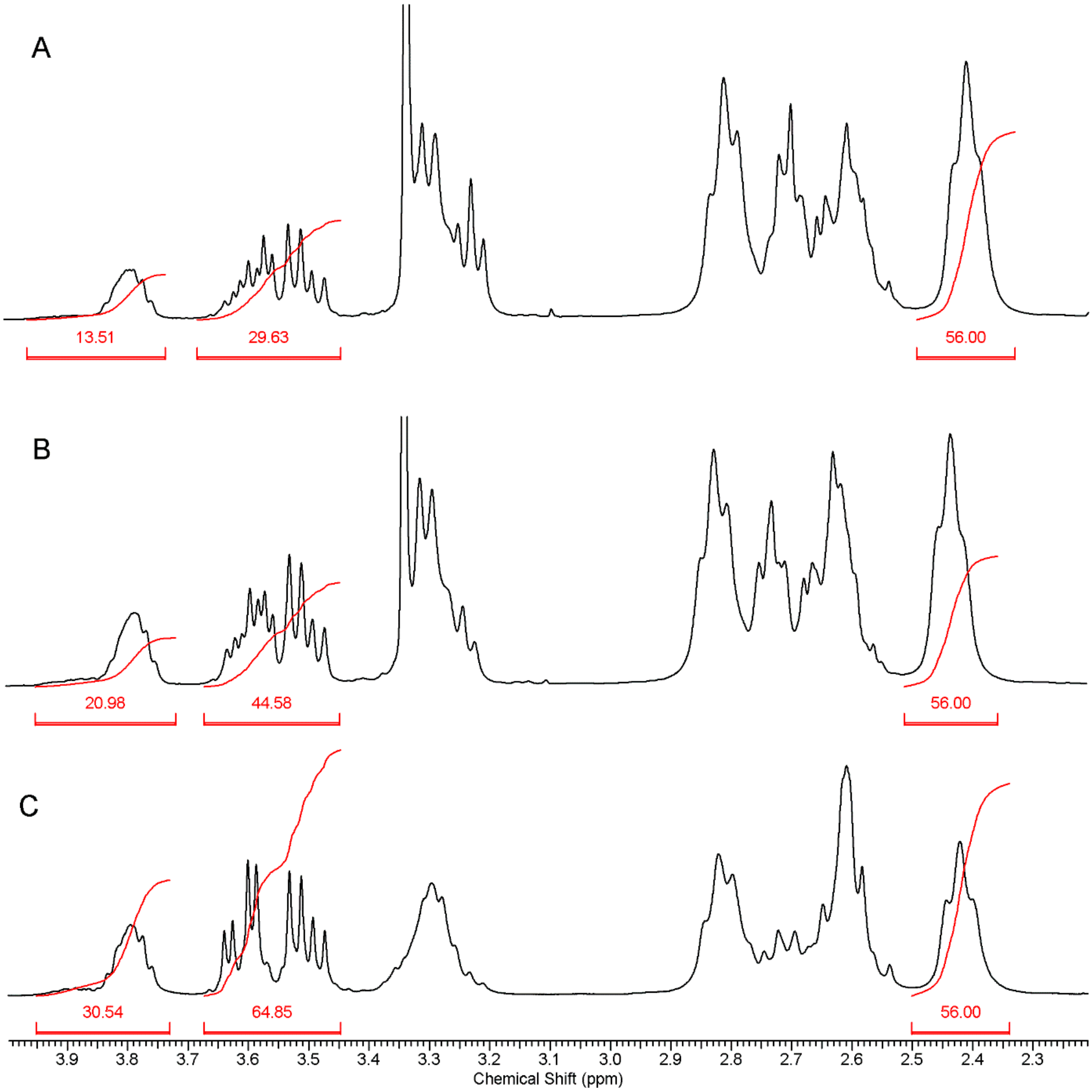

Figure S2. The ^{1}H NMR spectra of (**A**): **G2**^{14Rgl}, (**B**): **G2**^{22Rgl}, and(**C**): **G2**^{32Rgl} in D_2O.

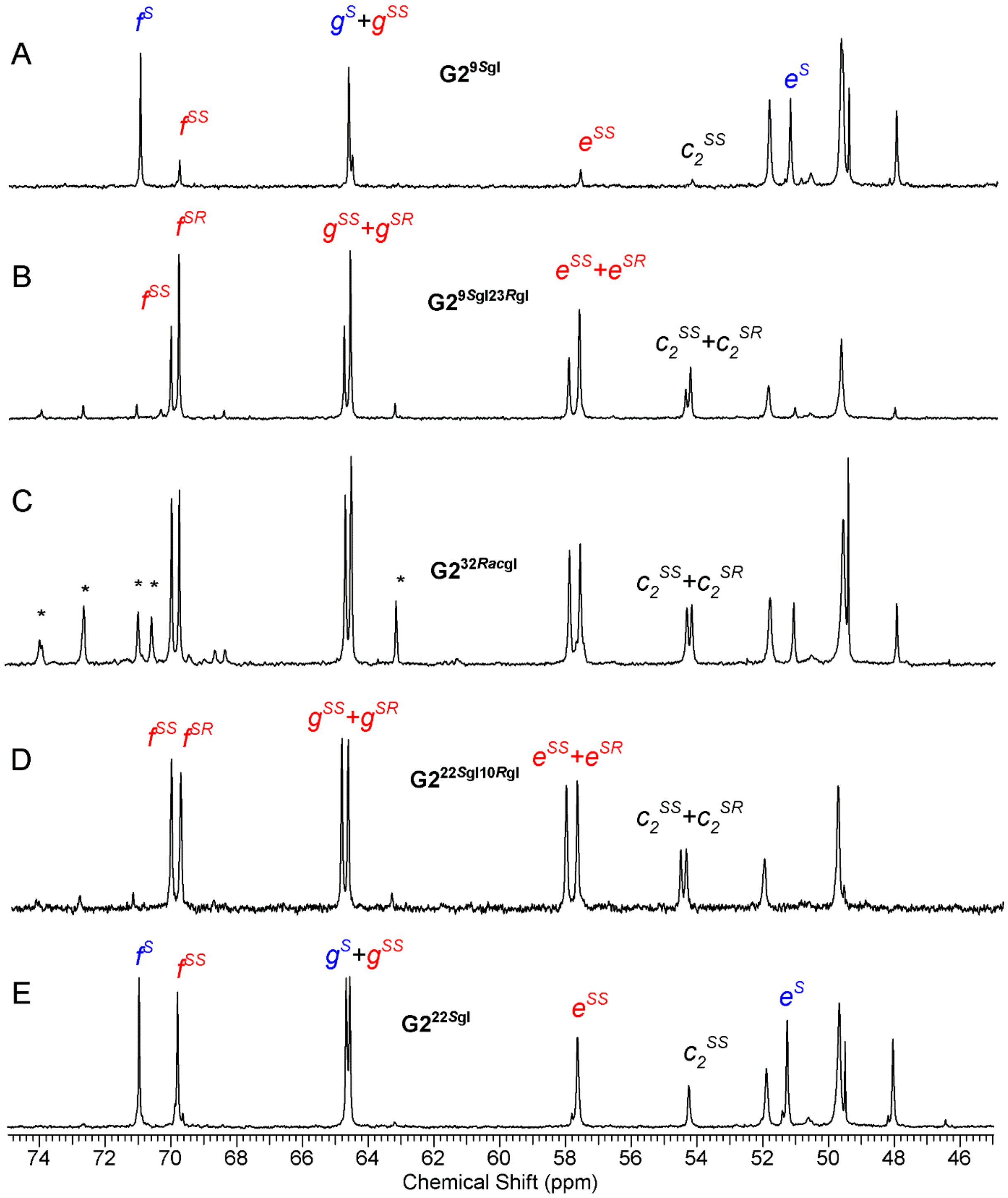

Figure S3. The relevant regions of ^{13}C NMR spectra (from top to bottom): (**A**): **G2**^{9Sgl}, (**B**): **G2**^{9Sgl23Rgl}, (**C**): **G2**^{32Racgl}, (**D**): **G2**^{22Sgl10Rgl}, and (**E**): **G2**^{22Sgl} in D_2O. For peak labeling see section 3.1.2.
